# Unbalanced Expression of Structural Genes in Carotenoid Pathway Contributes to the Flower Color Formation of the *Osmanthus* Cultivar ‘Yanzhi Hong’

**DOI:** 10.3390/ijms251810198

**Published:** 2024-09-23

**Authors:** Min Zhang, Zi-Han Chai, Cheng Zhang, Lin Chen

**Affiliations:** 1 Co-Innovation Center for Sustainable Forestry in Southern China, College of Life Sciences, Nanjing Forestry University, Nanjing 210037, China; czh31512451@163.com (Z.-H.C.); chengz@njfu.edu.cn (C.Z.); clinechen@njfu.edu.cn (L.C.); 2 International Cultivar Registration Center for Osmanthus, Nanjing Forestry University, Nanjing 210037, China

**Keywords:** transcription factor, LC-MS, transcriptome, WGCNA, *Osmanthus fragrans*

## Abstract

Carotenoids are important natural pigments that are responsible for the fruit and flower colors of many plants. The composition and content of carotenoid can greatly influence the color phenotype of plants. However, the regulatory mechanism underling the divergent behaviors of carotenoid accumulation, especially in flower, remains unclear. In this study, a new cultivar *Osmanthus fragrans* ‘Yanzhi Hong’ was used to study the regulation of carotenoid pigmentation in flower. Liquid chromatograph–mass spectrometer (LC-MS) analysis showed that *β*-carotene, phytoene, lycopene, *γ*-carotene, and lutein were the top five pigments enriched in the petals of ‘Yanzhi Hong’. Through transcriptome analysis, we found that the expression of the structural genes in carotenoid pathway was imbalanced: most of the structural genes responsible for lycopene biosynthesis were highly expressed throughout the flower developmental stages, while those for lycopene metabolism kept at a relatively lower level. The downregulation of *LYCE,* especially at the late developmental stages, suppressed the conversion from lycopene to *α*-carotene but promoted the accumulation of *β*-carotene, which had great effect on the carotenoid composition of ‘Yanzhi Hong’. Ethylene response factor (ERF), WRKY, basic helix-loop-helix (bHLH), v-myb avian myeloblastosis viral oncogene homolog (MYB), *N*-Acetylcysteine (NAC), auxin response factor (ARF), and other transcription factors (TFs) have participated in the flower color regulation of ‘Yanzhi Hong’, which formed co-expression networks with the structural genes and functioned in multiple links of the carotenoid pathway. The results suggested that the cyclization of lycopene is a key link in determining flower color. The modification of the related TFs will break the expression balance between the upstream and downstream genes and greatly influence the carotenoid profile in flowers, which can be further used for creating colorful plant germplasms.

## 1. Introduction

**Figure 1:**
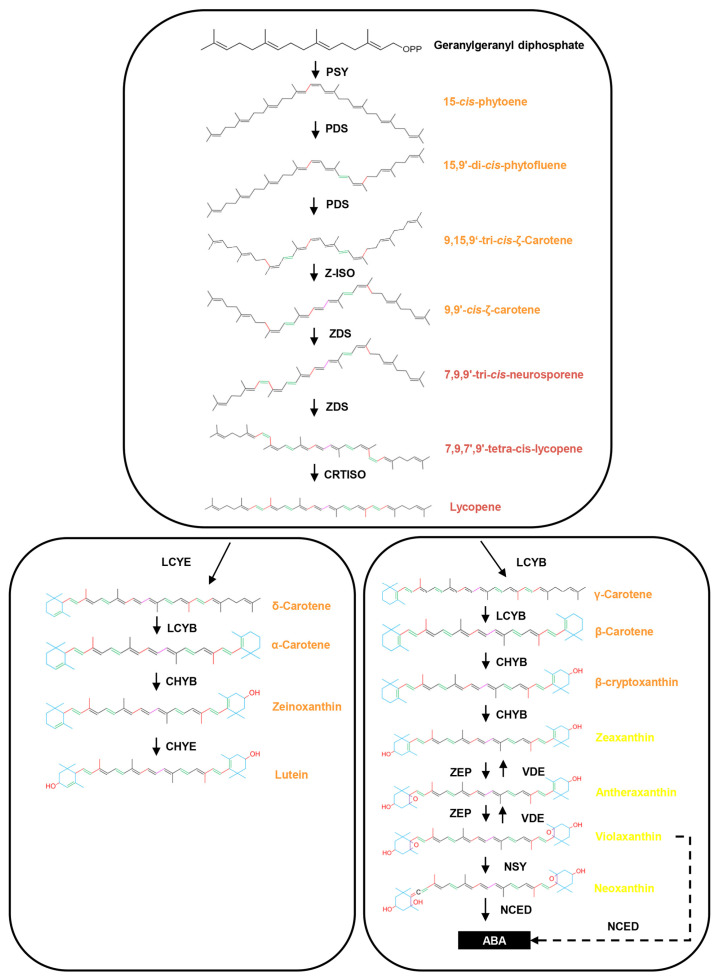
An illustration of the carotenoid biosynthesis pathway. Text color represents the color of the pigment.

Flower color is one of the most important features of angiosperm. The reason why flowers show different colors is mainly due to the variety of pigments and their relative contents. In plants, the pigments determining follower color can be divided into three major classes, including flavonoids, carotenoids, and betalains [1]. Among these metabolites, carotenoids contain more than 1000 members are the second most abundant natural pigments [2], which give flowers yellow to orange hues [3]. Specifically, xanthophylls and epoxy xanthophylls, such as zeaxanthin, antheraxanthin, violaxanthin, and neoxanthin, can impart pale yellow color to flowers; while lutein, *β*-cryptoxanthin, and carotenes always color flowers deep yellow to orange (Figure 1) [4]. Although carotenoids vary within a narrow color range, they are an important source of flower color variation. Many plants characterized by yellow and orange flowers are rich in carotenoids, and there is a considerable diversity in the carotenoid profiles, both within and between species [4]. In some plants, carotenoid accumulation even varies throughout the flowering stages and exhibits temporal features [5], which further enriches the diversity of flower colors in nature.

Over the years, a series of studies has been conducted to reveal the molecular mechanism underlying the divergent behaviors of carotenoid accumulation [6,7]. A highly conserved pathway of carotenoid biosynthesis and degradation, as well as the core genes and enzymes, has been well elucidated (Figure 1) [8,9,10]. Many transcription factors (TFs) involved in the regulation of carotenoid metabolism, such as WRKY [11], *N*-Acetylcysteine (NAC) [12,13], v-myb avian myeloblastosis viral oncogene homolog (MYB) [14], and basic helix-loop-helix (bHLH) [15], have also been identified. Now, we know that the divergence of carotenoid accumulation is largely determined by the differential expression of the structural genes and related TFs [10,16]. It seems that TFs establish a tight and precise control of gene expression along the carotenoid pathway, allowing for biosynthesis and catabolism to occur in accordance with specific requirements. However, current information about the transcriptional regulation of carotenoid is mainly derived from fruit. Very few TFs regulating carotenoid synthesis and metabolism have been identified in carotenoid-pigmented flowers [16]. It seems that fruits and flowers each have their own ways for regulating carotenoid [17]. In this case, our understanding of the transcriptional regulation of carotenoid biosynthesis and metabolism, especially in flowers, remains fragmented and incomplete [16]. Therefore, the investigation of transcriptional regulation of carotenoid metabolic genes remains the focus in uncovering the regulatory control of carotenoid accumulation in flower plants [10].

**Figure 2:**
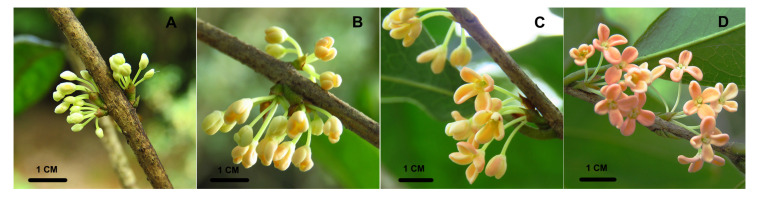
Flower developmental stages of *O. fragrans* ‘Yanzi Hong’ used in this study: (**A**) xiangyan stage (S1), (**B**) initial flowering stage (S2), (**C**) full flowering stage (S3), and (**D**) late full flowering stage (S4).

*Osmanthus fragrans*, belonging to the family Oleaceae, is one of the traditional top ten famous flowers in China, with more than 2500 years of cultivation [18]. During the long-term domestication, three cultivar groups with distinct flower colors (i.e., the Albus group, the Luteus group, and the Aurantiacus group) have been developed [19]. Specifically, the flower color of the Albus group is white to pale yellow, while the Luteus and Aurantiacus groups always have yellow and orange/red flowers, respectively [19]. Previous studies showed that the presence of white, yellow, and orange/red color varieties within *O. fragrans* was primarily attributable to the profile of carotenoids, especially *α*-carotene and *β*-carotene [20,21]. In the Albus and Luteus groups, *carotenoid cleavage dioxygenase 4* (*OfCCD4*) was significantly upregulated compared with that in the Aurantiacus group, which resulted in a higher efficiency level of carotenoid cleavage and relative lower content of *α*-carotene and *β*-carotene [20,22]. OfWRKY3, acting as an important positive regulator, might account for the overexpression of *OfCCD4* in the Albus and Luteus groups [20,22]. In addition, a 34-bp deletion mutation was found at a least one allele of *OfCCD4* in the Aurantiacus group [23], so the degradation of *β*-carotene in this group was partially or completely interrupted. However, these findings are still not enough to explain the dramatic flower color polymorphism within *O. fragrans*. Here, a new cultivar named *O. fragrans* ‘Yanzhi Hong’ was used to further study the flower color regulation of *O. fragrans* [24]. Unlike traditional cultivars, the flower color of ‘Yanzhi Hong’ is coccineous (Figure 2), making it an ideal material for the study of flower color variation. Through metabolome and transcriptome sequencing of the petals of ‘Yanzhi Hong’ at different developmental stages, we want to reveal the dynamic flower coloration processes, the regulatory network, and to enrich the theory of flower color regulation in plants.

## 2. Results

### 2.1. Carotenoid Accumulation in the Flowers of ‘*Yanzi Hong*’

**Figure 3:**
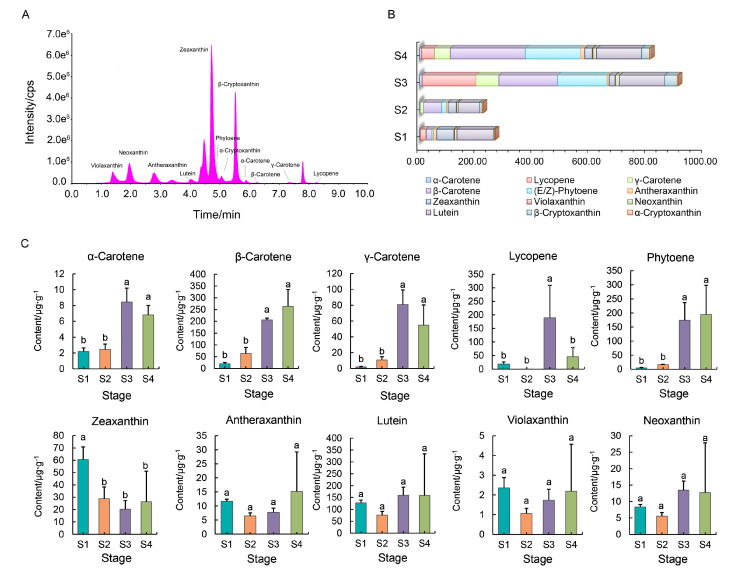
The dynamic composition and content of carotenoids in ‘Yanzhi Hong’. (**A**) Total ions chromatogram showing the appearance time of different carotenoids. (**B**) Changes in the composition and total content of carotenoids in ‘Yanzhi Hong’. (**C**) Histogram showing the difference in the carotenoid content across different developmental stages: S1, xiangyan stage; S2, initial flowering stage; S3, full flowering stage; S4, late full flowering stage. Different letters above bars indicate significant differences at the 0.05 level.

As shown in Figure 2, the petal color of ‘Yanzhi Hong’ changes with flower development. According to previous studies, carotenoids were the main pigments determining the flower color of *O. fragrans*. To reveal the global accumulation profile of carotenoids in the flowers of ‘Yanzhi Hong’, the content and composition of carotenoids in the petals at four developmental stages were investigated using the UPLC-APCI-MS/MS system. In total, 12 carotenoids, including *α*-carotene, *β*-carotene, *γ*-carotene, (E/Z)-phytoene, lycopene, antheraxanthin, zeaxanthin, violaxanthin, neoxanthin, lutein, *α*-cryptoxanthin, and *β*-cryptoxanthin were detected (Figure 3A). The accumulation of total carotenoids was slow at the early two developmental stages. After that, the total carotenoid content progressively increased (Figure 3B). The major carotenoid presented in the petals at the xiangyan stage (S1) was lutein, which accounted for 48.08% of total carotenoids, followed by zeaxanthin (22.80%), *β*-carotene (7.58%), and lycopene (7.12%). For the initial flowering stage (S2), the main carotenoids were lutein (33.93%), *β*-carotene (28.64%), zeaxanthin (12.90%), (E/Z)-phytoene (7.33%), and *γ*-carotene (4.82%), respectively. At the full flowering stage (S3), the top five carotenoids were *β*-carotene (22.68%), lycopene (20.81%), (E/Z)-phytoene (19.16%), lutein (17.51%), and *γ*-carotene (8.89%), respectively, while at the late full flowering stage (S4), they were, in turn, *β*-carotene (32.53%), (E/Z)-phytoene (23.89%), lutein (19.51%), *γ*-carotene (6.74%), and lycopene (5.64%).

During the whole flower developmental processes, different carotenoids exhibited divergent accumulation profiles (Figure 3C). The contents of *α*-carotene, *β*-carotene, *γ*-carotene, and (E/Z)-phytoene were continuously increased, and there was a significant difference between the first and the last two stages. However, unlike the other three pigments, α-carotene presented at low level throughout the flower development stages. Lycopene maintained a low level at stages S1 and S2; after that, it increased rapidly and reached its peak at stage S3. Zeaxanthin had the highest level of content at stage S1, then dropped significantly. The contents of antheraxanthin, violaxanthin, neoxanthin, lutein, *α*-cryptoxanthin, and *β*-cryptoxanthin in the petals of ‘Yanzi Hong’ were stable, and there was no significant divergence among different developmental stages.

### 2.2. Transcriptome Sequencing

To reveal the genes and TFs driving pigment accumulation, transcriptomes of the flowers from four developmental stages of ‘Yanzhi Hong’ were sequenced using the Illumina NovaSeq platform, respectively. In total, 513.50 Mb clean reads were obtained after data filtering, with a mean of 42.79 Mb (Table S1). The Q20 percentage of the data for each sample was over 95.21%, and the Q30 percentage was over 89.20%. The clean reads were then aligned to the reference genome. Finally, 35,644 genes were detected as expressed in the flower tissues.

### 2.3. Expression Patterns of Flower Color-Related Structural Genes

**Figure 4:**
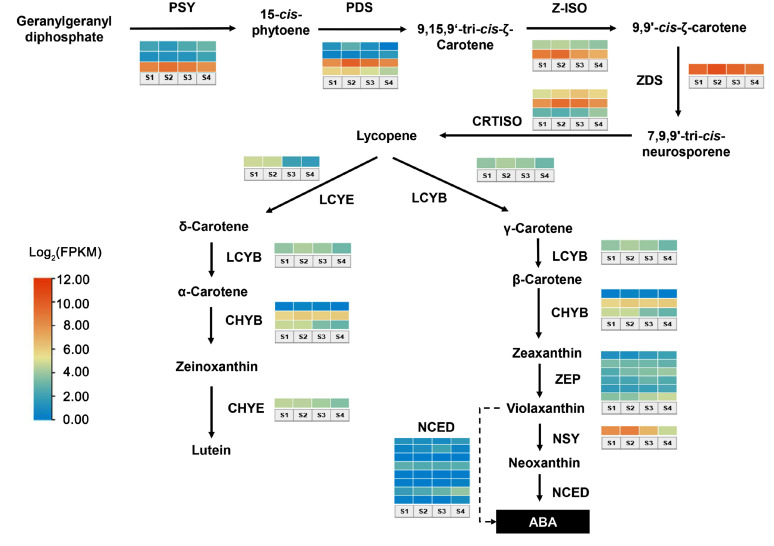
Heatmap showing the temporal expression patterns of key structural genes of carotenoid biosynthesis pathway in ‘Yanzhi Hong’. The colors of the blocks show the expression profile of different copies of the key genes, and each row represents one member of the structural gene.

The key genes involved in carotenoid biosynthesis and degradation were screened from the genome and transcriptome datasets, and heatmaps were used to illustrate the expression patterns of the structural genes during flower coloration, based on their FPKM values (Figure 4). The results showed that most structural genes involved in lycopene biosynthesis exhibited a higher level of expression during the flowering process compared to those involved in metabolism. Although with many members, at least one copy of *phytoene synthase* (*PSY*) and *15-cis phytoene desaturase* (*PDS*) were highly expressed in the petals of ‘Yanzhi Hong’. *Zeta-carotene isomerase* (*Z-ISO*), *zeta-carotene desaturase* (*ZDS*), and *carotenoid isomerase* (*CRTISO*), as three core genes coding enzymes catalyzing the desaturation reactions from 15-*cis*-phytoene to lycopene kept at a relatively high expression level throughout the studied stages. Conversely, the transcription abundance of nearly all the structural genes responsible for lycopene metabolism, including *lycopene e-cyclase* (*LCYE*), *lycopene b-cyclase* (*LCYB*), *P450-type β-carotene hydroxylase* (*CYP97A*, *CHYB*), *ε-hydroxylase* (*CHYE*), *zeaxanthin epoxidase* (*ZEP*), and *9-cis-epoxycarotenoid dioxygenase* (*NCED*), was relatively low.

**Figure 5:**
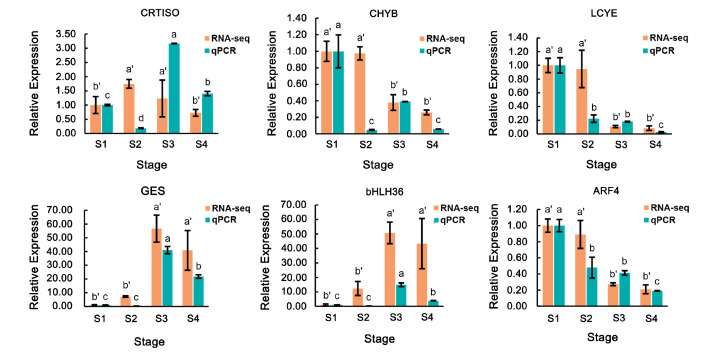
Quantitative analysis validating the results generated from RNA-seq. Different letters above bars indicate significant differences at the 0.05 level.

The temporal expression profile of the structural genes was also identified in this study. As shown in Figure 4, most of the highly expressed structural genes exhibited features of time series. The expression of *PSY*, *PDS*, *Z-ISO*, *ZDS*, and *CRTISO* increased at the first two stages and reached the peak at S2, then decreased gradually. The same pattern was also found in some genes responsible for *α*-carotene and *β*-carotene biosynthesis, as well as degradation, such as *LCYB*, *CHYB,* and *NSY*. *CHYE,* coding the key enzymes that catalyze lutein biosynthesis from zeinoxanthin, were downregulated over time. Some copies of *ZEP* and *NCED* were gradually upregulated at the late development stages but were limited at a low expression level. Most strikingly, the expression of *LCYE* maintained a relatively high level at the first two stages, then decreased dramatically. To confirm the authenticity of transcriptome sequencing data, the expression level of some core structural genes, including *CRTISO*, *CHYB*, and *LCYE,* was verified by qRT-PCR. The results showed that the transcript level of *CRTISO* at stage S3 was much higher than that at other stages, while *CHYB* and *LCYE* were downregulated over time, which was basically consistent with the results revealed by RNA-seq (Figure 5).

### 2.4. Identification of Gene Co-Expression Modules

**Figure 6:**
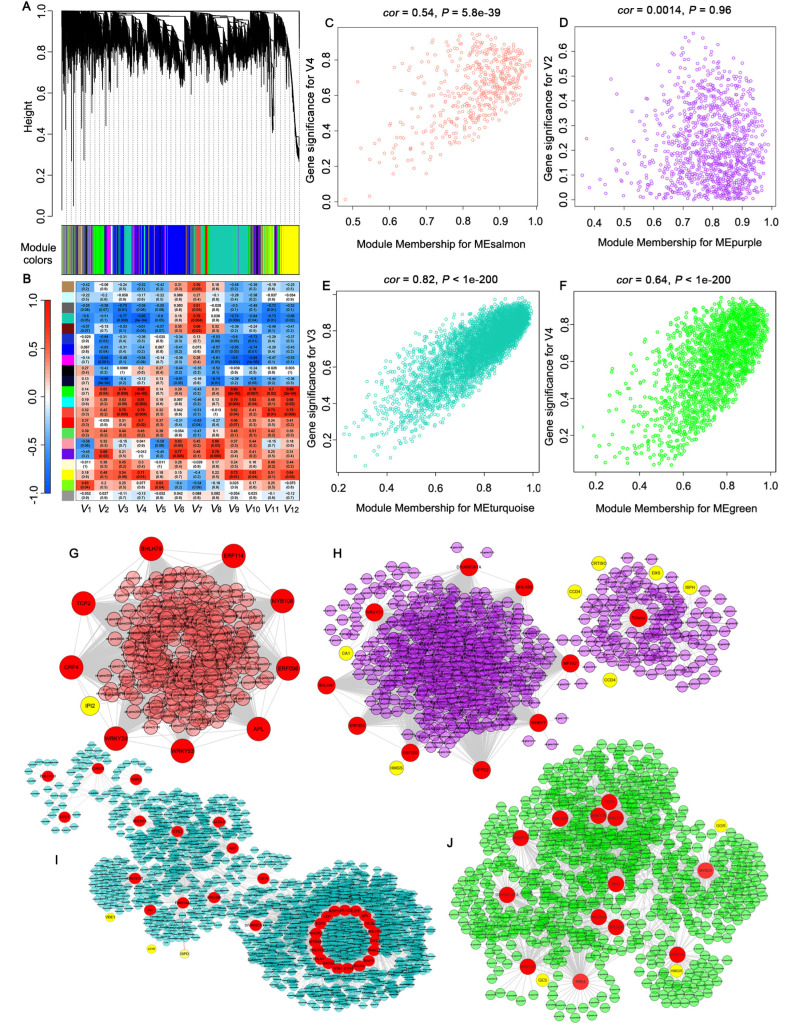
Co-expression network derived from WGCNA analysis. (**A**) Dendrogram of expressed genes clustered based on the measurement of dissimilarity (1-TOM). (**B**) Heatmap showing the correlation between module eigengenes and carotenoid content. *V1*–*V12* represent α-carotene, lycopene, *γ*-carotene, *β*-carotene, phytoene, antheraxanthin, zeaxanthin, violaxanthin, neoxanthin, lutein, *β*-cryptoxanthin, and *α*-cryptoxanthin, respectively. Scatterplot of gene significance for carotenoid content vs module membership in the (**C**) salmon module, (**D**) green module, (**E**) turquoise module, and (**F**) purple module; the correlation coefficient and *p*-value are listed above the scatterplots. (**G**–**J**) Co-expression relationship of TFs and the structural genes in the (**G**) salmon module, (**H**) purple module, (**I**) turquoise module, and (**J**) green module.

To investigate the gene regulatory network associated with flower color variation in ‘Yanzhi Hong’, gene clustering and module division were conducted through WGCNA. Modules were defined as clusters of highly interconnected genes, with genes within the same cluster showing high correlation coefficients in expression. After gene clustering, 35,644 genes were divided into 21 co-expression modules (Figure 6A). The number of genes in the modules ranged from 37 (royal blue) to 6639 (turquoise). Then, an eigengenes–trait correlation analysis was performed to reveal the relationship between module eigengenes and phenotypic traits. The results showed that the salmon, purple, turquoise, and green modules were significantly correlated with carotenoid accumulation in ‘Yanzhi Hong’ (*r*^2^ > 0.70, *P* < 0.01) (Figure 6B). For example, the salmon module was positively related to *γ*-carotene (V3), *β*-carotene (V4), *β*-cryptoxanthin (V11), and *α*-cryptoxanthin (V12). The purple module was positively associated with lycopene (V2) (*P* = 0.02), antheraxanthin (V6), and violaxanthin (V8). The turquoise module was negatively related to *γ*-carotene (V3), *β*-carotene (V4), and neoxanthin (V9), while positively related to zeaxanthin (V7). In particular, the green module was significantly positively associated with five kinds of carotenoids, including *γ*-carotene (V3), *β*-carotene (V4), neoxanthin (V9), lutein (V10), and *α*-cryptoxanthin (V12).

A KEGG enrichment analysis was performed to reveal the main functions of each module. Kegg terms such as “carotenoid biosynthesis” and “terpenoid backbone biosynthesis” were enriched from the salmon, purple, turquoise, and green modules, which were correlated with carotenoid accumulation in ‘Yanzhi Hong’. For the salmon module, *photosystem II D2* (*psbP*) was detected in the term “carotenoid biosynthesis”. *Isopentenyl-diphosphate Delta-isomerase II* (*IPI2*), *Protein-S-isoprenylcysteine O-methyltransferase* (*ICMTB*), and *Isoprene synthase* (*ISPS*) were detected in the term “terpenoid backbone biosynthesis”. *Beta-carotene hydroxylase 1* (*CA1*), *PDS*, *CRTISO*, and *carotenoid cleavage dioxygenase 4* (*CCD4*) were the main members in the “carotenoid biosynthesis” term of the purple module. *HMGS*, *4-hydroxy-3-methylbut-2-enyl diphosphate reductase* (*ISPH*), and *1-deoxy-D-xylulose-5-phosphate synthase* (*DXS*) were the main members of “terpenoid backbone biosynthesis”. In the turquoise module, *LCYE*, *violaxanthin de-epoxidase* (*VDE1*), *Z-ISO*, and *PDS* were enriched in the term “carotenoid biosynthesis”, while *geranylgeranyl pyrophosphate synthase* (*GGPS1*), *3-hydroxy-3-methylglutaryl-coenzyme A reductase* (*HMGCR*), *isopentenyl-diphosphate Delta-isomerase II* (*IDI2*), *1-deoxy-D-xylulose 5-phosphate reductoisomerase* (*DXR*), *phosphomevalonate kinase* (*PMK*), *2-C-methyl-D-erythritol 4-phosphate cytidylyltransferase* (*ISPD*) were enriched in the term “terpenoid backbone biosynthesis”. When it comes to the green module, only *zeaxanthin epoxidase* (*ABA2*) was identified in the term “carotenoid biosynthesis”, while six genes of *GGR*, *geraniol synthase* (*GES*), *CAAX prenyl protease 1* (*FACE1*), *3-Hydroxy-3-methylglutaryl-CoA reductase* (*HMGR*), *farnesyl pyrophosphate synthase 1* (*FPS1*), and *HMGCR* were involved in “terpenoid backbone biosynthesis”.

### 2.5. Identification of Transcription Factors Related to Flower Color Regulation

Key transcription factors were identified from the main co-expression modules associated with carotenoid accumulation (Figure 6G–J). In total, 19 TFs, belonging to the families of MYB, proliferating cell factor (TCP), WRKY, auxin response factor (ERF), and bHLH, were identified from the salmon module. GATA-type transcription factor (GATA), ERF, MYB, bHLH, WRKY, NAC, and DIVARICATA were the main transcription factors in the purple and turquoise modules, with a total number of 150 and 45, respectively. There were 90 TFs, mainly involved in bHLH, ERF, WRKY, GATA, NAC, MYB, and DIVARICATA families in the green module. Network analysis was further performed to identify the key TFs which have high connectivity with other genes. As shown in Figure 6G, WRKY28, ERF098, MYB108, APL, ERF114, TCP2, bHLH79, WRKY53, and cytokinin response factor 4 (CRF4) were the main TFs in the salmon module, with a high degree of connection to the structural genes, of which CRF4 and WRKY28 had a relatively higher connectivity with *IPI2*. For the purple module, the trihelix transcription factor (ASIL1), WRKY21, ERF025, ERF003, WRKY7, DIVARICATA, bHLH80, nuclear transcription factor Y subunit gamma 2 (NFYC2), and Trihelix, as main TFs, had wide connections with *ISPH*, *HMGS*, *CRTISO*, *DXS*, *CCD4*, *CA1*, and other genes related to carotenoid accumulation (Figure 6H). In the turquoise module, GATA9, E2FE, MYB44, bHLH130, bHLH25, bHLH130, EMB1444, DIVARICATA, NAC056, and other TFs formed a complex regulatory network and intertwined with *VDEs*, *LCYE,* and *ISPD* (Figure 6I). When it comes to the green module, WRKY70, MYB23, ILI1 binding bHLH protein1 (IBH1), DIVARICATA, TCP9, WRKY23, WRKY41, PRE4, and bHLH36 exhibited higher connectivity with other genes, and they were either directly or indirectly related to *GES*, *HMGR*, and *GGR*, which encode key enzymes in the carotenoid pathway (Figure 6J).

## 3. Discussion

### 3.1. Main Pigments Determining Flower Color of ‘*Yanzhi Hong*’

Recent studies have revealed that carotenoids, especially *α*-carotene and *β*-carotene, play an import role in the flower color formation of *O. fragrans* [20,21], and the divergent metabolism efficiency of *β*-carotene is an important source of flower color variation among different cultivar groups [22,23]. However, until now over 200 *Osmanthus* varieties with different colors have been documented [19]. Therefore, the divergence of *β*-carotene metabolism only is far from enough to explain the dramatic flower color polymorphism within *O. fragrans*. In this study, *O. fragrans* ‘Yanzhi Hong’ was used to study the flower color regulation of *O. fragrans*. Unlike the traditional cultivars, the flower color of ‘Yanzhi Hong’ was light yellow at the early stages, then turned coccineous (Figure 2). We used LC-MS analysis to reveal the main pigments degerming the flower color of this special cultivar. The results showed that *β*-carotene, (E/Z)-phytoene, lycopene, *γ*-carotene, and lutein were the top five pigments enriched in the petals of ‘Yanzhi Hong’ (Figure 3). Specifically, the contents of *β*-carotene and (E/Z)-phytoene continuously increased and reached their peaks at S4. Though there was no significant difference in components between S4 and S3, an upward trend was observed. Lycopene kept at a low level at stages S1 and S2, then increased rapidly (Figure 3). The accumulation profile of the above pigments was basically consistent with the dynamic flower color change of ‘Yanzhi Hong’. For carotenoids, *β*-carotene, phytoene, and lutein always exhibit deep yellow to orange hues, but lycopene is coccineous (Figure 1). Based on these facts, it can be concluded that *β*-carotene, (E/Z)-phytoene, *γ*-carotene, and lutein provided the base color of ‘Yanzhi Hong’, while lycopene acted as the matching color and contributed to the flower color transformation from yellow to coccineous at the late developmental stages. The final color phenotype of ‘Yanzhi Hong’ is the combined effect of the above pigments.

Lycopene is a natural pigment synthesized exclusively by plants and microorganisms. When synthesized, there are two ways for lycopene to be metabolized: one pathway leads to *α*-carotene, while the other leads to *β*-carotene (Figure 1). Unlike the traditional *Osmanthus* cultivars [20,21], *α*-carotene remained at a low level throughout the flower development stages of ‘Yanzhi Hong’, indicating its weak effect on color formation. It seemed that the conversion from lycopene to *α*-carotene was inefficient, although the precursor used for *α*-carotene synthesis was sufficient, as lycopene was highly accumulated at the late developmental stages (Figure 3). In contrast, *γ*-carotene and *β*-carotene were continuously accumulated. Obviously, more lycopene was used for *β*-carotene synthesis rather than *α*-carotene in ‘Yanzhi Hong’. These signs indicated that the metabolism of lycopene was a crucial link determining the flower color of ‘Yanzhi Hong. Since the biosynthesis of *α*-carotene was suppressed, lycopene could only flow into *β*-carotene, resulting in a surplus at the late developmental stages, which painted the flowers coccineous. It should also be noted that the downstream products, including lutein, zeaxanthin, neoxanthin, antheraxanthin, and violaxanthin, did not show significant difference among different stages (Figure 3). This means that the metabolism of *α*-carotene or *β*-carotene could proceed normally, so the downstream products could be accumulated steadily and maintain the base color of ‘Yanzhi Hong’.

### 3.2. Unbalanced Expression of Structural Genes Contributes to the Carotenoids Profile of ‘*Yanzhi Hong*’

The biosynthesis and metabolism of carotenoids rely on a series of enzymes. To investigate the key structural genes that drive carotenoid accumulation in ‘Yanzhi Hong’, we analyzed the whole expression profile of genes through the pathway by using RNA-seq. We found that the overall transcription abundance of the upstream and downstream genes in ‘Yanzhi Hong’ was imbalanced. Specifically, most of the structural genes involved in the biosynthesis of lycopene remained at a relatively higher level of expression throughout the flower developmental stages (Figure 4). This can be seen from *PSY*, *PDS*, *Z-ISO*, *ZDS*, and *CRTISO*. In contrast, the expression level of nearly all the structural genes responsible for lycopene metabolism, including *LCYE*, *LCYB*, *CHYB*, *ZEP*, and *NCED*, was relatively lower. The imbalanced expression of the aforementioned structural genes directly determined the carotenoid accumulation profile of ‘Yanzhi Hong’. During flower development, phytoene and lycopene were accumulated continuously in ‘Yanzhi Hong’ (Figure 3). This was due, on one hand, to the high expression of *PSY*, *PDS*, *Z-ISO*, *ZDS*, and *CRTISO*, which encode critical enzymes and ensure that the synthesis reactions are carried out efficiently, and on the other, to the relatively low and stable consumption of downstream reactions. In ‘Yanzhi Hong’, the expression level of *LCYE*, *LCYB*, *CHYB*, *ZEP*, and *NCED* was relatively low, and the overall demand of lycopene was limited accordingly. In addition, the biosynthesis of *α*-carotene from lycopene was suppressed to some extent, which further affected the metabolic efficiency of lycopene, causing it to become surplus in the later developmental stages.

Based on the transcriptome data, we also revealed the temporal expression features of structural genes in the carotenoid pathway. The expression level of most genes, including *PSY*, *PDS*, *Z-ISO*, *ZDS*, *CRTISO*, *LCYB*, *CHYB,* and *NSY*, was increased with flower development and reached their peak at stages S2 or S3, then decreased to some extent (Figure 4 and Figure 6). This is consistent with the law of plant growth and development. However, the downregulation of overall gene expression does not imply a reduction in carotenoid content, since pigment accumulation is the result of three independent processes: carotenoid biosynthesis, degradation, and stable storage [8,25]. Though the expression of the structural genes decreased at the late developmental stages, the synthesis and storage of the substances continued. Therefore, we can see many pigments, especially *β*-carotene and phytoene, obtained their top content at stage S4 (Figure 3). It is noteworthy that the expression level of *LYCE* was extremely low at stages S3 and S4 (Figure 4). That may be the key reason for the inefficient biosynthesis of *α*-carotene from lycopene in ‘Yanzhi Hong’. In plants, lycopene represents the branch point of the carotenoid pathway because it acts as the substrate for two competing enzymes, *LYCB* and *LYCE* [26]. For ‘Yanzhi Hong’, *LYCE* was not dominant, and the cyclization of lycopene in the late stages of flower development was mainly catalyzed by *LYCB*. In this case, the biosynthesis of *α*-carotene was suppressed, but the consumption was not reduced, so its content was low. This was different from the gene expression pattern revealed in other *O. fragrans* cultivars. Previous studies concluded that the divergent expression of *CCD4* was responsible for the flower color variation within *O. fragrans* [22,23]. However, our results showed that the flower color of ‘Yanzhi Hong’ was mainly determined by the metabolism of lycopene, and the downregulation of *LYCE*-induced inefficient lycopene metabolism played an important role in the formation of this special cultivar. We believe that carotenoid composition is an important source of flower color variation since different carotenoids always display different colors, as shown in Figure 1. There may be several key links that directly affect carotenoid composition in plants, including the cleavage of *α*-carotene and *β*-carotene by *CCD4,* as described by previous studies [20,22,27,28], and the cyclization of lycopene by *LYCE* and *LCYB,* as revealed in this study and in other research studies [29,30]. An unbalanced or divergent expression of genes encoding crucial essential enzymes in these links will greatly influence carotenoid composition, which can be further used for creating colorful plant germplasms.

### 3.3. Potential Transcription Factors Regulating Carotenoid Accumulation in ‘*Yanzhi Hong*’

As described above, the expression divergence of structural genes is always associated with a series of transcription factors. Many TFs, including WRKY, NAC, MYB, and bHLH, were reported to have participated in the regulation of carotenoid pigmentation [11,12,13,14,15,16]. However, current information about the transcriptional regulation of carotenoids is mainly derived from fruit-related studies. Very few TFs regulating carotenoid synthesis and metabolism have been identified in carotenoid-pigmented flowers [16]. What is more, a study on tomato showed that none of the regulators involved in fruit coloration dramatically affect flower petal color [17], which indicates that fruits and flowers have different approaches in carotenoid biosynthesis regulation. Therefore, uncovering TFs and their regulatory control of carotenoid accumulation in flowers should be an important direction for future research [10]. *O. fragrans* provides an ideal material for the study of flower color regulation, since the intraspecific variation of flower color is extremely abundant. In this study, based on transcriptome data and trait association analysis, we constructed the gene co-expression network during flower development in ‘Yanzhi Hong’. Many gene expression modules closely related to carotenoid accumulation have been identified (Figure 6). In the modules, ERF, WRKY, bHLH, MYB, NAC, GATA, TCP, DIVARICATA, and ARF exhibited a high degree of connection with other genes and acted as important nodes of the co-expression networks. Since the modules were significantly associated with carotenoid content, we speculated that these TFs also played important roles in regulating carotenoid accumulation in the flower of ‘Yanzhi Hong’. It seemed that there were internal links among these TFs, and they worked together as modules rather than individually to regulate carotenoid accumulation, which is why the crucial TFs revealed by different studies were always divergent. In addition, each TF family contained a lot of members with different expression patterns, which made the co-expression networks even more complicated.

To further explore the potential TFs that determine the flower color of ‘Yanzhi Hong’, we analyzed the co-expression relationship of TFs and the structural genes in each module. In the green module, TFs such as WRKY70, WRKY23, WRKY41, MYB23, DIVARICATA, and bHLH36, combined with the structural genes *GES*, *HMGR*, *ISPS,* and *GGR*, formed a co-expression network which was positively associated with the *γ*-carotene, *β*-carotene, neoxanthin, lutein, *β*-cryptoxanthin, and *α*-cryptoxanthin contents (Figure 6), and they were continuously upregulated throughout the development stages (Figure 5 and Figure S1). *GES*, *HMGR*, *ISPS*, and *GGR* were key genes in the terpenoid backbone biosynthesis pathway, encoding enzymes that catalyze the precursor synthesis of carotenoids [31]. We thought that these TFs could promote the accumulation of *γ*-carotene, *β*-carotene, lutein, and other carotenoids in ‘Yanzhi Hong’ by accelerating the biosynthesis of the precursors of lycopene. In contrast, GATA9, E2FE, MYB44, bHLH44, bHLH25, bHLH130, ARF4, NAC056, and other TFs, intertwined with *VDEs*, *LCYE*, *CHYB,* and *ISPD,* formed the turquoise module, which was negatively related to the *γ*-carotene and *β*-carotene contents. According to previous studies, bHLH [32], NAC [33], and MYB [34,35] always act as positive regulators of carotenoid biosynthesis. However, when it comes to the turquoise module in this study, they were continuously downregulated (Figure 5 and Figure S1), which is inconsistent with the law of carotenoid accumulation. We speculate that the turquoise module was responsible for *α*-carotene biosynthesis and *β*-carotene metabolism. The downregulation of the above TEs, on one hand, acted on *LCYE*, resulting in its low expression and ultimately leading to the inefficient conversion from lycopene to *α*-carotene; on the other hand, it acted on *CHYB*, inhibiting the degradation of *β*-carotene. Therefore, we can see the different accumulation profiles of *α*-carotene and *β*-carotene in ‘Yanzhi Hong’. *CCD4* is a crucial gene in the flower color formation of *O. fragrans* that has received much attention in previous studies. Downregulation of *OfCCD4* resulted in a relative low level of *α*-carotene and *β*-carotene content in the Aurantiacus group [20,22]. In this work, two copies of the functional *CCD4* (ofr.gene22473 and ofr.gene22483) in the purple module were highly expressed (Log_2_FPKM > 8) through the flower developmental stage of ‘Yanzhi Hong’ (Figure S2B), and they were significantly upregulated compared to the other copies. This means that the cleavage of *β*-carotene by *CCD4* was not restricted, and *CCD4* was not the key factor determining the content of *β*-carotene in ‘Yanzhi Hong’. A previous study on *O. fragrans* found that OfWRKY was able to bind to the promoter of *OfCCD4* [36]. In our work, ERF025, ERF003, WRKY7, WRKY21, DIVARICATA, bHLH80, NFYC2 were key TFs in the purple module, and they had wide connections with *ISPH*, *HMGS*, *DXS* and *CRTISO*, in addition to *CCD4s*. *ISPH*, *HMGS*, and *DXS* were key genes in the terpenoid backbone biosynthesis pathway [31], while *CRTISO* and *CCD4s* belong to the carotenoid biosynthesis pathway [10]. Therefore, these TFs might act at different steps along the carotenoid pathway. The same pattern could also be seen at the salmon module (Figure 6). We predict that these modules play important roles in balancing the upstream and downstream reactions during carotenoid accumulation.

## 4. Materials and Methods

### 4.1. Plant Materials

The materials used in this study were collected from Jinhua City, Zhejiang Province, China (119°38′42.57″ E, 28°58′15.51″ N). According to previous studies [20], the flower development process of *O. fragrans* is divided into four stages (Figure 2), namely, the xiangyan stage (S1), the initial flowering stage (S2), the full flowering stage (S3), and the late full flowering stage (S4). Flowers from the four stages of ‘Yanzhi Hong’ were harvested and frozen in liquid nitrogen immediately, then transferred to a −80 °C freezer for storage until RNA extraction. For component extraction, flowers from each stage were freeze-dried, then stored at −80 °C until needed.

### 4.2. Chemicals and Reagents

Methanol, ethanol, and acetonitrile were purchased from Merck (Darmstadt, Germany). BHT (Butylated hydroxytoluene) was purchased from Aladdin (Shanghai, China). Acetone was purchased from Sinopharm (Beijing, China). Methyl tert-butyl ether (MTBE) was purchased from CNW (Shanghai, China). NaCl and KOH were purchased from Rhawn (Shanghai, China) and Hushi (Shanghai, China), respectively. MilliQ water (Millipore, Bradford, PA, USA) was used in all experiments. All the standards were purchased from Olchemim Ltd. (Olomouc, the Czech Republic) and Sigma (St. Louis, MO, USA). Formic acid was obtained from Sigma and BOC (New York, NY, USA). Stock solutions of the standards were prepared at the concentration of 1 mg/mL. All stock solutions were stored at −20 °C.

### 4.3. Sample Preparation and Carotenoid Extraction

The dried flowers were homogenized and powdered in a mill. Then 50 mg of dried powder was extracted with mixed solution of n-hexane/acetone/ethanol (1:1:2, *v*/*v*) and extra 0.01% BHT (g/mL). Then, the internal standards were added. The extract was vortexed for 20 min at room temperature. The supernatants were collected after centrifugation. To the supernatants, a saturated sodium chloride solution was added and vortexed, and the upper layer was collected. Next, the supernatant was evaporated to dryness and dissolved in MTBE, then 10% KOH-MeOH was added. The mixture was vortexed, allowing the reaction to take place at room temperature overnight. After the reaction, a saturated sodium chloride solution was added and vortexed, the upper layer was collected, and the supernatant was evaporated to dryness and reconstituted in mixed solution of methanol/MTBE (3:1, *v*/*v*). Finally, the solution was filtered through a 0.22 μm filter for further LC-MS analysis.

### 4.4. UPLC Conditions

Carotenoids in the flowers of ‘Yanzhi Hong’ were detected using an UPLC-APCI-MS/MS system (UPLC, ExionLC™ AD, https://sciex.com.cn/, accessed on 15 December 2023; MS, Applied Biosystems 6500 Triple Quadrupole, https://sciex.com.cn/, accessed on 15 December 2023). The analytical conditions were as follows: LC: column, YMC C30 (3 μm, 100 mm × 2.0 mm i.d); solvent system, methanol/acetonitrile (1:3, *v/v*) with 0.01% BHT and 0.1% formic acid (A), methyl tert-butyl ether with 0.01% BHT (B); a gradient program that started at 0% B (3 min), increased to 70% B (5 min), then increased to 95% B (9 min), and finally ramped back to 0% B (10 min); flow rate, 0.8 mL/min; temperature, 28 °C; injection volume: 2 μL.

### 4.5. APCI-MS/MS Parameters

The APCI source operation parameters were as follows: ion source, APCI+; source temperature, 350°C; curtain gas (CUR), 25.0 psi. DP and CE for individual MRM transition were performed with further DP and CE optimization. A specific set of MRM transitions were monitored for each period according to the carotenoids eluted within this period. During the experimental process, the mixed standard solution was used as the quality control sample. A quality control sample was inserted every 10 test analysis samples during the instrument analysis process, allowing for the assessment of instrument stability during the project test by comparing the total ion flow chromatogram (TIC) of the same quality control sample through overlapping display analysis, along with the essential spectrum.

### 4.6. Standard Curve Drawing and Quantitative Analysis

Carotenoid standard solutions with different concentrations (0.001 μg/mL, 0.005 μg/mL, 0.01 μg/mL, 0.05 μg/mL, 0.1 μg/mL, 0.5 μg/mL, 1 μg/mL, 5 μg/mL, 10 μg/mL, 50 μg/mL, 100 μg/mL, 250 μg/mL, and 400 μg/mL) were prepared. The mass spectrum peak intensity data of the corresponding quantitative signals of the standard solution were obtained. Taking the concentration ratio between the external standard and the internal standard as the horizontal coordinate and the peak area ratio between the external standard and the internal standard as the longitudinal coordinate, the standard curves of different substances were drawn. The ratio of the integrated peak area of all samples detected was calculated by substituting it into the linear equation of the standard curve. This value was further calculated using the formula to obtain the absolute content of the substance in the actual sample.

### 4.7. RNA Extraction and Sequencing

Total RNA was extracted from the flowers of ‘Yanzhi Hong’ at four developmental stages using a RNAprep Puree Plant Kit DP441 (TIANGEN, Beijing, China) according to the manufacturer’s instructions, respectively. For each stage, three biologic replicates were set up. The quantity and quality of RNA were detected using a Nanodrop 1000 spectrophotometer (Thermo Fisher Scientific, Wilmington, DE, USA). The qualified RNA was then used for sequencing library construction. In total, 12 libraries were constructed, and then paired-end (2 × 150 bp) sequenced using the Illumina NovaSeq 6000 platform (Illumina Inc., San Diego, CA, USA) by Beijing Genomics Institute (BGI, Shenzhen, China) with the standard Illumina RNA-seq protocols.

### 4.8. Bioinformatics Process

High-quality clean reads were obtained by removing reads with adaptors, ambiguous reads with more than 5% unknown bases (N), and low-quality reads with more than 20% low-quality bases (quality value < 15) using the FastQC tool (http://www.bioinformatics.babraham.ac.uk/projects/fastqc/, accessed on 10 December 2023). The effective data were then mapped to the *O. fragrans* genome assembled by our laboratory using bowtie2 (version 2.1.0) [37]. Gene expression levels were estimated using the FPKM method by RSEM (version 1.3.0) [38], and only uniquely mapped reads were kept for FPKM calculation.

To further elucidate the regulatory mechanism of carotenoid accumulation in *O. fragrans* flowers, we constructed the co-expression networks using the WGCNA (v1.47) package in R [39]. The soft-threshold of co-expression network clustering was selected according to the FPKM values of all genes in the samples, with *R*^2^ > 0.8 as the norm. Genes with FPKM > 0 in at least nine samples were retained. A total of 35,644 genes were obtained for WGCNA. Different genes were categorized by the dynamic tree-cut method into distinct co-expression modules, and the minimum number of genes in each co-expression module was set to 30. The correlation between different modules and the degree of association (module membership, MM) of genes within the modules were calculated. The key modules related to flower color were found for subsequent analysis. Cytoscape_V.3.0.0 was used to visualize the regulatory networks.

### 4.9. Quantitative Real-Time PCR (qRT-PCR) Validation

Quantitative real-time PCR (qRT-PCR) was used to validate the results of RNA-seq. Gene-specific primer pairs were designed by Primer Premier software (version 5.0) according to the corresponding sequences (Table 1). A SYBR Green Premix Pro Taq HS qPCR Kit (AG11701, ACCURATE BIOTECHNOLOGY, HUNAN, Co., Ltd., Changsha, China) was used for real-time PCR reactions. The *OfACT* gene was chosen as the internal reference gene [40]. A relative quantitative method (delta–delta Ct) was used to evaluate the relative expression level of candidate genes. All qRT-PCR experiments included three individuals for each accession and three technical replicates for each individual.

## 5. Conclusions

In this study, the regulation of flower color in plants is revealed based on studies conducted on *O. fragrans* ‘Yanzhi Hong’. Through metabolome analysis, we found that *β*-carotene, phytoene, lycopene, *γ*-carotene, and lutein were the main pigments enriched in the petals of ‘Yanzhi Hong’. Unlike other *Osmanthus* cultivars, lycopene also played an important role in the flower color formation of this special cultivar. Transcriptome analysis showed that the expression of the structural genes in the carotenoid pathway was imbalanced. Most of the structural genes responsible for lycopene biosynthesis were highly expressed throughout the flower developmental stages, while those responsible for lycopene metabolism remained at a relatively lower level. In particular, the downregulation of *LYCE* at the late developmental stages suppressed the conversion from lycopene to *α*-carotene but promoted the accumulation of *β*-carotene, which had a great effect on the carotenoid composition of ‘Yanzhi Hong’. ERF, WRKY, bHLH, MYB, NAC, ARF, and other TFs participated in the flower color regulation of ‘Yanzhi Hong’. They formed co-expression networks with the structural genes and functioned in multiple links of the carotenoid pathway. Our results revealed that the cyclization of lycopene is a key link determining flower color. The modification of the aforementioned TFs will break the balance between the upstream and downstream genes and greatly influence the carotenoid profile, which can be further used for creating colorful plant germplasms.

## Figures and Tables

**Table 1:** Primer pairs used in this study.

No.	GeneID	Annotation	Forward Primer (5′-3′)	Reverse Primer (5′-3′)
1	ofr.gene19681	*CRTISO*	ATGCCTTGAACTCGCTGGAA	CGGCAGATAGTAGGCAAGGG
2	ofr.gene3952	*CHYB*	AAATGGGCGTTGGGTGGTAA	TTCTCCACACTCTGTACGCC
3	ofr.gene42137	*LCYE*	GGTGGATCCTTGCCGAGTAC	TAACCTGTGGCTGGATGGAC
4	ofr.gene54192	*GES*	AAGGAGGAGGAAGAACGAGG	ATGGCATTGCGTTTGGTGAA
5	ofr.gene24362	*ARF4*	GTGACTGGAGTTGGTGACGT	TGGCAGACTTGATCGCAGTT
6	ofr.gene39242	*bHLH36*	AAGAAGGTGCTGAGGTCCTC	GTGCAGACGTCTCTTCAAGC
7	ofACT [40]	*Actin*	CCCAAGGCAAACAGAGAAAAAAT	ACCCCATCACCAGAATCAAGAA

## Data Availability

All the sequencing data are available in the NCBI Sequence Read Archive (SRA) database with the BioProject accession number of PRJNA1044907.

## Supplementary Materials

The following supporting information can be downloaded at: https://www.mdpi.com/article/10.3390/ijms251810198/s1.

## References

[1] Mol J., Grotewold E., Koes R. (1998). How genes paint flowers and seeds. Trends Plant Sci..

[2] Yabuzaki J. (2017). Carotenoids database: Structures, chemical fingerprints and distribution among organisms. Database.

[3] Tanaka Y., Sasaki N., Ohmiya A. (2008). Biosynthesis of plant pigments: Anthocyanins, betalains and carotenoids. Plant J..

[4] Ohmiya A. (2011). Diversity of carotenoid composition in flower petals. Jpn. Agric. Res. Q..

[5] Guo L., Wang Y., da Silva J.A.T., Fan Y., Yu X. (2019). Transcriptome and chemical analysis reveal putative genes involved in flower color change in *Paeonia* ‘Coral Sunset’. Plant Physiol. Biochem..

[6] Hao Z., Liu S., Hu L., Shi J., Chen J. (2020). Transcriptome analysis and metabolic profiling reveal the key role of carotenoids in the petal coloration of *Liriodendron tulipifera*. Hort. Res..

[7] Pu X., Li Z., Tian Y., Gao R., Hao L., Hu Y., He C., Sun W., Xu M., Peters R.J. (2020). The honeysuckle genome provides insight into the molecular mechanism of carotenoid metabolism underlying dynamic flower coloration. New Phytol..

[8] Nisar N., Li L., Lu S., Khin N.C., Pogson B.J. (2015). Carotenoid metabolism in plants. Mol. Plant.

[9] Hermanns A.S., Zhou X., Xu Q., Tadmor Y., Li L. (2020). Carotenoid pigment accumulation in horticultural plants. Hortic. Plant J..

[10] Sun T., Li L. (2020). Toward the ‘golden’ era: The status in uncovering the regulatory control of carotenoid accumulation in plants. Plant Sci..

[11] Chen H., Ji H., Huang W., Zhang Z., Zhu K., Zhu S., Chai L., Ye J., Deng X. (2024). Transcription factor CrWRKY42 coregulates chlorophyll degradation and carotenoid biosynthesis in *Citrus*. Plant Physiol..

[12] Fu C.C., Han Y.C., Fan Z.Q., Chen J.Y.Y., Chen W.X.X., Lu W.J., Kuang J.F. (2016). The papaya transcription factor CpNAC1 modulates carotenoid biosynthesis through activating phytoene desaturase genes *CpPDS2/4* during fruit ripening. J. Agric. Food Chem..

[13] Xing S., Li R., Zhao H., Zhai H., He S., Zhang H., Zhou Y., Zhao N., Gao S., Liu Q. (2023). The transcription factor IbNAC29 positively regulates the carotenoid accumulation in sweet potato. Hort. Res..

[14] Song J., Sun B., Chen C., Ning Z., Zhang S., Cai Y., Zheng X., Cao B., Chen G., Jin D. (2023). An R-R-type MYB transcription factor promotes non-climacteric pepper fruit carotenoid pigment biosynthesis. Plant J..

[15] Zhou D., Shen Y., Zhou P., Fatima M., Lin J., Yue J., Zhang X., Chen L.Y., Ming R. (2019). Papaya *CpbHLH1/2* regulate carotenoid biosynthesis-related genes during papaya fruit ripening. Hort. Res..

[16] Stanley L., Yuan Y.W. (2019). Transcriptional regulation of carotenoid biosynthesis in plants: So many regulators, so little consensus. Front. Plant Sci..

[17] Galpaz N., Ronen G., Khalfa Z., Zamir D., Hirschberg J. (2006). A chromoplast-specific carotenoid biosynthesis pathway is revealed by cloning of the tomato white-flower locus. Plant Cell.

[18] Zang D.K., Xiang Q.B. (2004). Studies on *Osmanthus fragrans* cultivars. J. Nanjing Forestry Uni..

[19] Xiang Q., Liu Y. (2007). An Illustrated Monograph of the Sweet Osmanthus Variety in China.

[20] Han Y., Wang X., Chen W., Dong M., Yuan W., Liu X., Shang F. (2014). Differential expression of carotenoid-related genes determines diversified carotenoid coloration in flower petal of *Osmanthus fragrans*. Tree Genet. Genomes.

[21] Wang Y., Zhang C., Dong B., Fu J., Hu S., Zhao H. (2018). Carotenoid accumulation and its contribution to flower coloration of *Osmanthus fragrans*. Front. Plant Sci..

[22] Han Y., Wu M., Cao L., Yuan W., Dong M., Wang X., Chen W., Shang F. (2016). Characterization of OfWRKY3, a transcription factor that positively regulates the carotenoid cleavage dioxygenase gene *OfCCD4* in *Osmanthus fragrans*. Plant Mol. Biol..

[23] Chen H., Zeng X., Yang J., Cai X., Shi Y., Zheng R., Wang Z., Liu J., Yi X., Xiao S. (2021). Whole-genome resequencing of *Osmanthus fragrans* provides insights into flower color evolution. Hort. Res..

[24] Xiang Q., Wang X., Liu Y. (2014). Annual report ICRCO 2014 (2) three new cultivars of *Osmanthus fragrans*. J. Nanjing Forestry Uni..

[25] Li L., Yuan H. (2013). Chromoplast biogenesis and carotenoid accumulation. Arch. Biochem. Biophys..

[26] Peng A., Tang X., Feng Y., Huang Y., Cui J., Tian K., Lu M., Zhao Y., Pan Y., Wang S. (2023). Molecular mechanism of lycopene cyclases regulating carotenoids ratio in different branches during tea leaf and flower development. Hortic. Plant J..

[27] Wen L., Wang Y., Deng Q., Hong M., Shi S., He S., Huang Y., Zhang H., Pan C., Yang Z. (2020). Identifying a carotenoid cleavage dioxygenase (*CCD4*) gene controlling yellow/white fruit flesh color of “Piqiutao” (white fruit flesh) and its mutant (yellow fruit flesh). Plant Mol. Biol. Rep..

[28] Li T., Deng Y.J., Liu J.X., Duan A.Q., Liu H., Xiong A.S. (2021). *DcCCD4* catalyzes the degradation of *α*-carotene and *β*-carotene to affect carotenoid accumulation and taproot color in carrot. Plant J..

[29] Bai L., Kim E.H., DellaPenna D., Brutnell T.P. (2009). Novel lycopene epsilon cyclase activities in maize revealed through perturbation of carotenoid biosynthesis. Plant J..

[30] Wang Y.H., Zhang Y.Q., Zhang R.R., Zhuang F.Y., Liu H., Xu Z.S., Xiong A.S. (2023). Lycopene *ε*-cyclase mediated transition of *α*-carotene and *β*-carotene metabolic flow in carrot fleshy root. Plant J..

[31] Vranová E., Coman D., Gruissem W. (2013). Network analysis of the MVA and MEP pathways for isoprenoid synthesis. Annu. Rev. Plant Biol..

[32] Liu Z., Mao L., Yang B., Cui Q., Dai Y., Li X., Chen Y., Dai X., Zou X., Ou L. (2023). A multi-omics approach identifies bHLH71-like as a positive regulator of yellowing leaf pepper mutants exposed to high-intensity light. Hort. Res..

[33] Zhu M., Chen G., Zhou S., Tu Y., Wang Y., Dong T., Hu Z. (2014). A new tomato NAC (NAM/ATAF1/2/CUC2) transcription factor, SlNAC4, functions as a positive regulator of fruit ripening and carotenoid accumulation. Plant Cell Physiol..

[34] Sagawa J.M., Stanley L.E., LaFountain A.M., Frank H.A., Liu C., Yuan Y.W. (2015). An R2R3-MYB transcription factor regulates carotenoid pigmentation in *Mimulus lewisii* flowers. New Phytol..

[35] Xi W., He Y., Zhu L., Hu S., Xiong S., Zhang Y., Zou J., Chen H., Wang C., Zheng R. (2021). CPTA treatment reveals potential transcription factors associated with carotenoid metabolism in flowers of *Osmanthus fragrans*. Hortic. Plant J..

[36] Liu Y., Zhou B., Qi Y., Chen X., Liu C., Liu Z., Ren X. (2017). Expression differences of pigment structural genes and transcription factors explain flesh coloration in three contrasting kiwifruit cultivars. Front. Plant Sci..

[37] Langmead B., Salzberg S.L. (2012). Fast gapped-read alignment with Bowtie 2. Nat. Methods.

[38] Li B., Dewey C.N. (2011). RSEM: Accurate transcript quantification from RNA-Seq data with or without a reference genome. BMC Bioinform..

[39] Langfelder P., Horvath S. (2008). WGCNA: An R package for weighted correlation network analysis. BMC Bioinform..

[40] Zhang C., Fu J., Wang Y., Bao Z., Zhao H. (2015). Identification of suitable reference genes for gene expression normalization in the quantitative real-time PCR analysis of sweet osmanthus (*Osmanthus fragrans* Lour.). PLoS ONE.

