# Supplementary material for: Unbalanced Expression of Structural Genes in Carotenoid Pathway Contributes to the Flower Color Formation of the Osmanthus Cultivar ‘Yanzhi Hong’
Source: Int J Mol Sci. 2024 Sep 23;25(18):10198. doi: 10.3390/ijms251810198 (PMC11432492; doi:10.3390/ijms251810198)
Supplement: Supplementary file 1 [file ijms-25-10198-s001.zip › ijms-3202508-supplementary.pdf]

Table S1. Summary of transcriptome sequencing for the accessions in this study.

Sample	Total Raw Reads (M)	Total Clean Reads (M)	Clean Reads Q20(%)	Clean Reads Q30(%)	Clean Reads Ratio (%)
YZH1_1	47.33	42.52	95.53	89.84	89.84
YZH1_2	48.97	43.39	95.23	89.2	88.6
YZH1_3	47.33	43.05	95.46	89.64	90.96
YZH2_1	47.33	42.19	95.26	89.27	89.15
YZH2_2	47.33	42.87	95.27	89.25	90.58
YZH2_3	45.57	41.98	95.41	89.53	92.11
YZH3_1	47.33	43.04	95.2	89.23	90.94
YZH3_2	47.33	42.94	95.42	89.7	90.73
YZH3_3	47.33	42.35	95.35	89.58	89.49
YZH4_1	49.08	43.45	95.21	89.25	88.54
YZH4_2	47.33	43.51	95.25	89.21	91.94
YZH4_3	47.33	42.21	95.59	89.94	89.18

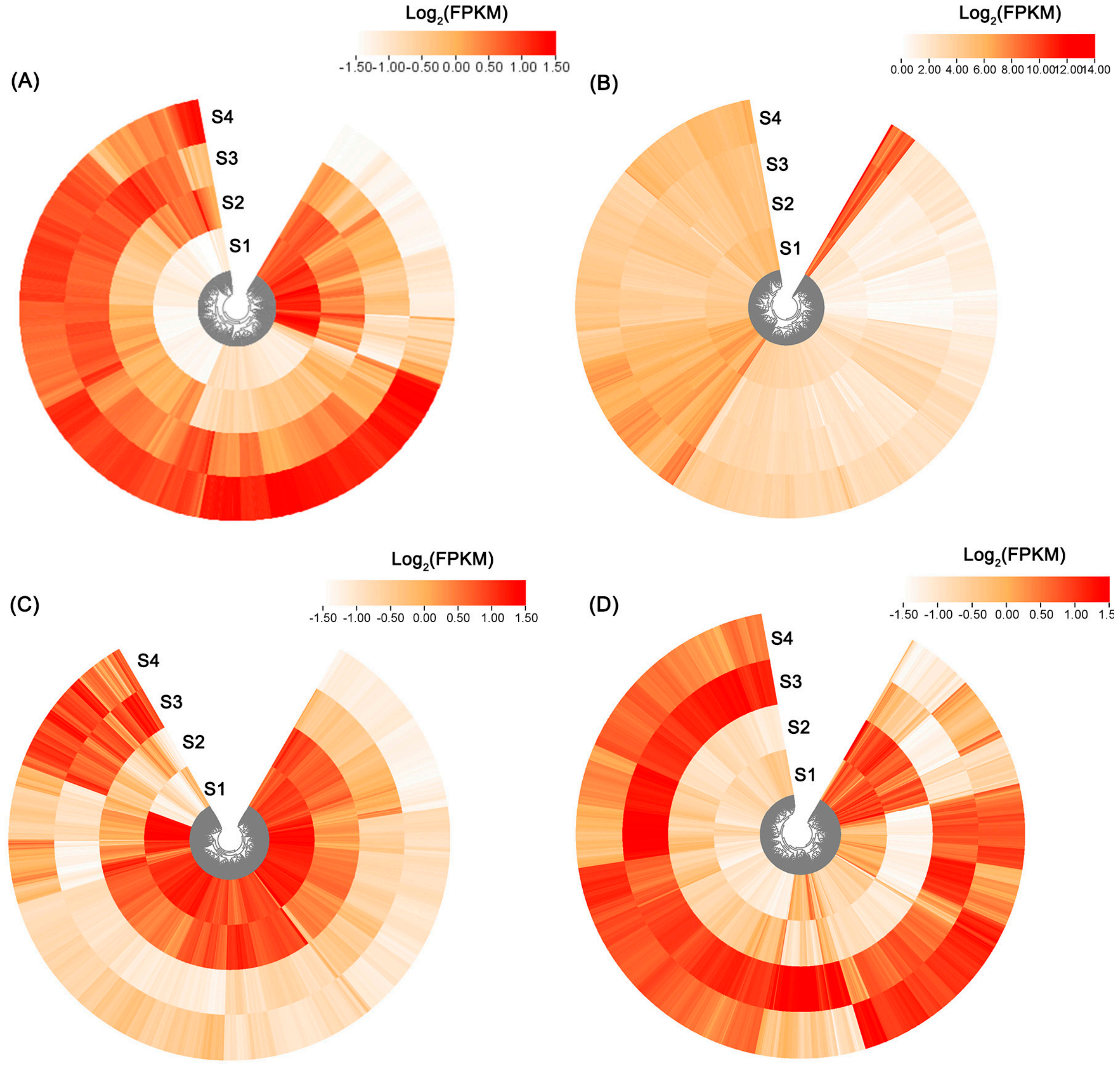

Figure S1. Heatmap showing the gene expression pattern of the modules related to flower color of 'Yanzhi Hong'. (**A**) salmon module, (**B**) purple module; (**C**) turquoise module and (**D**) green module. S1-S4 indicating the four development stages of xiangyan stage (S1), initial flowering stage (S2), full flowering stage (S3), and late full flowering stage (S4), respectively.

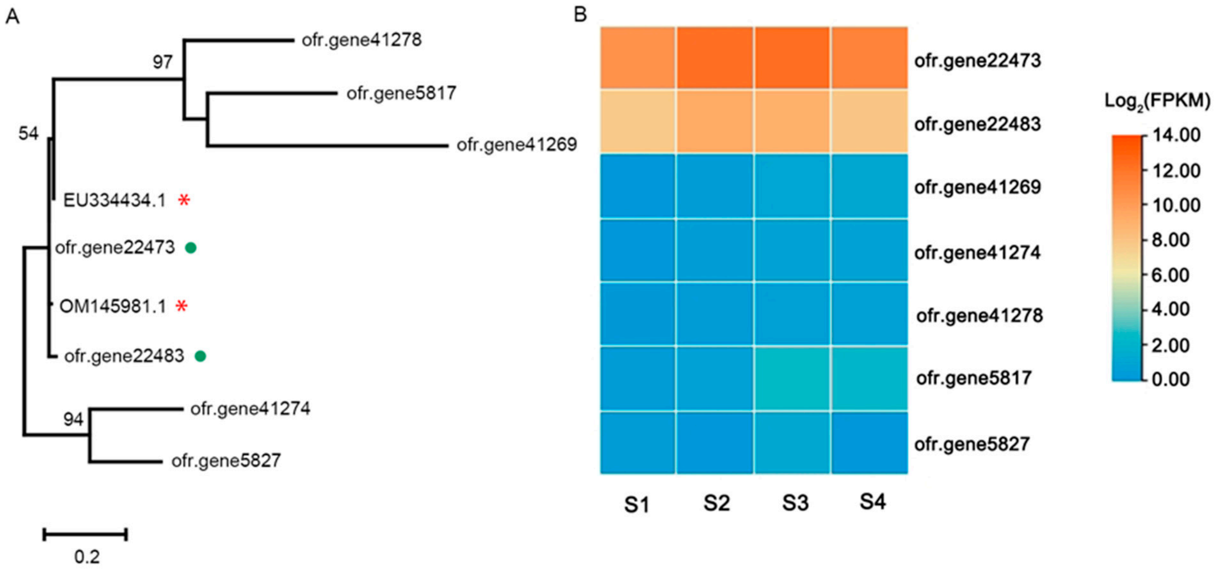

Figure S2. The expression profile of different *CCD4* members in 'Yanzhi Hong'. (**A**) ML tree showing the relationship of different *CCD4* members expressed in 'Yanzhi Hong'. The * marks the known functional *CCD4* sequences of *O. fragrans* from genbank. The green solid circles show the two homologous genes of the functional *CCD4* expressed in 'Yanzhi Hong'. (**B**) Heatmap showing the overall gene expression of *CCD4*s in 'Yanzhi Hong'. S1-S4 indicating the four-development stage of xiangyan stage (S1), initial flowering stage (S2), full flowering stage (S3), and late full flowering stage (S4), respectively.
